# Toward a Standardized Strategy of Clinical Metabolomics for the Advancement of Precision Medicine

**DOI:** 10.3390/metabo10020051

**Published:** 2020-01-29

**Authors:** Nguyen Phuoc Long, Tran Diem Nghi, Yun Pyo Kang, Nguyen Hoang Anh, Hyung Min Kim, Sang Ki Park, Sung Won Kwon

**Affiliations:** 1 College of Pharmacy, Seoul National University, Seoul 08826, Korea; phuoclong@snu.ac.kr (N.P.L.); 2018-23140@snu.ac.kr (N.H.A.); snuhmkim04@snu.ac.kr (H.M.K.); 2 Department of Life Sciences, Pohang University of Science and Technology, Pohang 790-784, Korea; diemnghi0208@postech.ac.kr (T.D.N.); skpark@postech.ac.kr (S.K.P.); 3 Department of Cancer Physiology, Moffitt Cancer Center and Research Institute, Tampa, FL 33612, USA; yunpyo.kang@moffitt.org

**Keywords:** adaptive metabolomics, lipidomics, multi-omics, precision medicine, systems biology, machine learning

## Abstract

Despite the tremendous success, pitfalls have been observed in every step of a clinical metabolomics workflow, which impedes the internal validity of the study. Furthermore, the demand for logistics, instrumentations, and computational resources for metabolic phenotyping studies has far exceeded our expectations. In this conceptual review, we will cover inclusive barriers of a metabolomics-based clinical study and suggest potential solutions in the hope of enhancing study robustness, usability, and transferability. The importance of quality assurance and quality control procedures is discussed, followed by a practical rule containing five phases, including two additional “pre-pre-” and “post-post-” analytical steps. Besides, we will elucidate the potential involvement of machine learning and demonstrate that the need for automated data mining algorithms to improve the quality of future research is undeniable. Consequently, we propose a comprehensive metabolomics framework, along with an appropriate checklist refined from current guidelines and our previously published assessment, in the attempt to accurately translate achievements in metabolomics into clinical and epidemiological research. Furthermore, the integration of multifaceted multi-omics approaches with metabolomics as the pillar member is in urgent need. When combining with other social or nutritional factors, we can gather complete omics profiles for a particular disease. Our discussion reflects the current obstacles and potential solutions toward the progressing trend of utilizing metabolomics in clinical research to create the next-generation healthcare system.

## 1. Introduction

Metabolomics offers a pragmatic and robust framework for the comprehensive measurement and identification of the endogenous and exogenous low-molecular-weight metabolites in biological systems [1]. Together with other omics platforms, e.g., genomics and proteomics, metabolomics offers a systematic assessment of the interactions between genetic variations, central metabolism, and environmental exposures to explain the initiation and progression of a disease [2]. It is understood that the causation in systems biology should determine how and why a process occurs in a certain way rather than identify separate molecular components [3]. In this regard, measuring and modeling the metabolome can shed light on the pathophysiological mechanisms and provide an essential piece of information for precision medicine.

In clinical medicine and epidemiology, together with the development of machine learning and artificial intelligence, omics analyses empower systems biology-based methods for precision health monitoring and treatment [4]. Typical applications of omics technologies in precision medicine encompass the assessment of biomarker panel to estimate disease risk, diagnose disease, quantify progression, and optimize treatment strategies [5]. Even so, the achievements of metabolomics in large-scale research are somewhat limited [6,7,8,9,10]. Up to the present time, a significant part of the metabolomics-based epidemiological and clinical studies is cross-sectional or non-prospective case-control, which compares the metabolic phenotypes of participants by disease or exposure status [11,12]. These study designs are intricate in establishing the temporal precedence and based on prevalent rather than incident cases; thus, they are rather the preliminary step of the process of diagnostic biomarker discovery. The majority of diagnostic biomarker discovery studies are conducted with the lack of external validation, leading to the high false-positive rates and insufficient data on the predictive value of biomarkers in pre-diagnostic samples [13]. The commonly applied strategies of metabolomics biomarkers in clinical and epidemiological studies are not adequate to provide sufficient predictive evidence to be clinically useful, as observed in the study aiming at identifying biomarkers for early pancreatic cancer diagnosis [14]. Other clinical fields, such as obstetrics, are also in urgent need of large-scale studies using a well-defined sampling strategy to determine predictive biomarkers for preterm birth given the current heterogeneity in methodologies and identification methods across different studies [15].

The Consortium of Metabolomics Studies has discussed experience gained from current epidemiological metabolomics studies worldwide and suggested guidelines concerning important aspects of a large-scale metabolomics study [16]. A recently published review from the Consortium of Metabolomics Studies Statistics Working Group delineated a framework involving study and bio-sampling design for studies that integrate metabolomics with other omics data as well as considerations on multi-omics data processing techniques at the population level [17]. Extending the boundaries of these perspectives, our review will cover all-inclusive barriers in a metabolomics study at the clinical and epidemiological scale, primarily focusing on mass spectrometry (MS)-based approaches. We will investigate analytical and computational challenges, thus proving the need for the establishment of a standardized biobank, quality assurance (QA) and quality control (QC) standards, the establishment of an automated system for sample handling and data acquisition, standards for metabolite and lipid identification, as well as the usability of machine learning. We will provide several discussions on fundamental requirements on standards for reporting metabolomics studies at the clinical and epidemiological scale. Significantly, automated systems of machine learning and artificial intelligence platforms are predicted to be developed soon and integrated into various steps of a metabolomics study [18]. Machine learning methods such as random forests and deep neural networks, which are reliable in prediction, have a “black box” nature, causing a substantial debate, the so-called “cascade of black box data manipulations, processing, and analyses” in omics research. The trade-off between model accuracy and interpretability can be regarded as an emerging crisis in the field. Collectively, we propose a comprehensive and adaptive multi-omics workflow demonstrating the indispensable position of metabolomics in the omics universe along with a proper reporting guideline. This practice, if deeply implemented, may potentially extend the translation of omics science into clinical settings for the next-generation healthcare system. Additionally, we have tried to raise current concerns that are open for active discussion throughout the paper.

## 2. Representative Achievements of Metabolomics in Public Health and Clinical Research

Following emerging instrumental and logistics technologies, analytical methods, and computational tools, research on the application of hydrophilic and lipophilic metabolite biomarkers in epidemiological studies has been increasing over the last decade, particularly on cancer, cardiovascular diseases, and diabetes, but it is still relatively sparse compared with other omics approaches [8]. MS is the primary analytical method on which the emerging omics fields such as proteomics and metabolomics are based. Owing to its potential as a multi-platform analytical method and its diverse applicability from diagnosis and prognosis to the discovery of novel biomarkers for targeted therapies, MS has been routinely applied in omics studies, especially cancer and metabolism-related omics research, for a better insight into human diseases [19,20,21,22,23,24,25,26]. Table 1 displays the main findings of some major studies among the initial achievements of the field.

The lack of independent validation cohorts remains challenging in most early metabolomics studies at population-based level, which is gradually being improved in later large-scale projects [41,42,43,44]. Prospective longitudinal studies with larger sample size, longer follow-up period, and more efficient nested design (e.g., nested case-control, case-cohort, and clinical trials) have been conducted recently to assist the biomarker establishment [11,45,46,47]. Despite being cost- and time-consuming, prospective study designs may serve both biomarker discovery and validation purposes and can be equipped with a standardized biobank as a platform for biomarker research to overcome the limitations of cross-sectional studies [13]. Future research can benefit from this model to provide clinically robust results and eventually help innovate the healthcare system.

## 3. Analytical and Computational Challenges in Clinical Metabolomics and Lipidomics Studies

**Figure 1:**
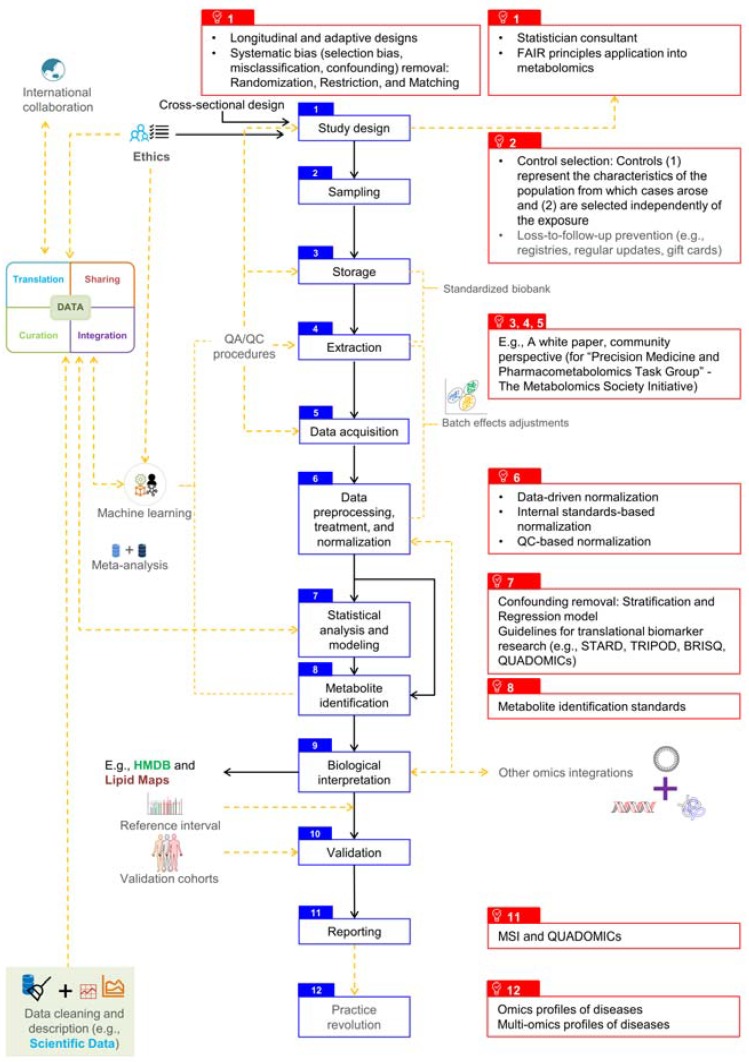
The standard untargeted metabolomics workflow at clinical and epidemiological scale. Blue boxes constitute the backbone of the workflow. Black arrows indicate current standards. Orange dashed arrows imply that the issues have been raised but have not received enough attention or have lacked communication. Red boxes represent current and/or potential solutions to these issues. BRISQ: Biospecimen Reporting for Improved Study Quality. FAIR: Findability, Accessibility, Interoperability, and Reusability. MSI: Metabolomics Standards Initiative. QUADOMICs: Quality Assessment of studies on the diagnostic accuracy of OMICs-based technologies. STARD: Standards for the Reporting of Diagnostic Accuracy Studies. TRIPOD: Transparent Reporting of a multivariable prediction model for Individual Prognosis or Diagnosis.

The general workflow of untargeted metabolomics research is described in Figure 1. Furthermore, large-scale multi-site studies require stringent awareness of systematic and random errors, batch variation, differences between analytical instruments, heterogeneity across centers regarding methods for storage, handling, and transportation [48]. We have made attempts to inspect and address these issues throughout this section sequentially. Particular challenges of lipidomics will also be discussed in detail.

### 3.1. The Need for Standardized Biobank Establishment

The preanalytical steps in sample acquisition and processing are among the most decisive for producing reliable analytical results [49]. Currently, it is challenging to collect and optimally handle samples because of the collaboration of multiple research laboratories and long shipping to main hubs and freezers [50]. It is also of importance to emphasize that the number of biobanks worldwide has experienced a rapid escalation, giving rise to the issue of interbiobank variability [51]. The majority of biobanks established for epidemiological purposes benefit either genomics or more traditional epidemiologic analyses, whereas the collection of DNA requires profoundly different criteria than that of metabolites. The metabolome is dynamically changing, and metabolism can continue in tissues or biofluids after collection even when being carried on with great care [49]. Collectively, the establishment of biobanks dedicated to metabolomics along with other omics is currently needed, and harmonization in the methods of sample collection, storage, analytical methods protocols should be defined within the study design to secure the requirement of reproducible research as well as reduce the cost of future research. Significant attempts have been made by International Agency for Research on Cancer Biobank, Promoting Harmonization of Epidemiological Biobanks in Europe, and Biobanking and Biomolecular Resources Research Infrastructure, for instance, on the way to reach a standardized, unified, fully validated protocol where all steps have been tested for reproducibility and robustness [52,53,54]. With that same goal, recommendations on the processing and biobanking of serum, plasma, tissues, urine, feces, as well as other biofluids have been discussed at great length in the paper of “Precision Medicine and Pharmacometabolomics Task Group” from the Metabolomics Standards Initiative (MSI) [49]. Furthermore, primary aspects of biobank harmonization have been comprehensively unveiled in this white paper.

Under adverse sample handling conditions and delayed freezing, only 62% of metabolites had a stable level and good to excellent concordance [55]. At a long-term epidemiological scale, however, the impact of different storage conditions on metabolite stability needs to be more profoundly examined since the plasma concentrations of specific metabolites can be altered under five-year storage at −80 °C [56]. Potential solutions to ensure sample stability in the long run would be the exclusion or better adjustment coefficient of profoundly altered compounds prior to the establishment of biomarker panel, development of more accurate analytical techniques for unstable but biologically essential metabolites, development of better long-term storage strategies such as liquid nitrogen storage, and avoidance of repeated freeze-thaw cycles. Efforts have been recorded; for instance, the European Federation of Clinical Chemistry and Laboratory Medicine Working Group on Preanalytical Phase is currently working on some recommendations for a study focusing on sample stability to enable standardization and reproducibility [57]. Another problem appears in case each protocol of the biobank has a different influence on the metabolite changes. Thus, research for better recommendations on how samples should be interpreted may be a potential topic.

### 3.2. The High Demand for Quality Assurance and Quality Control Standards

**Figure 2:**
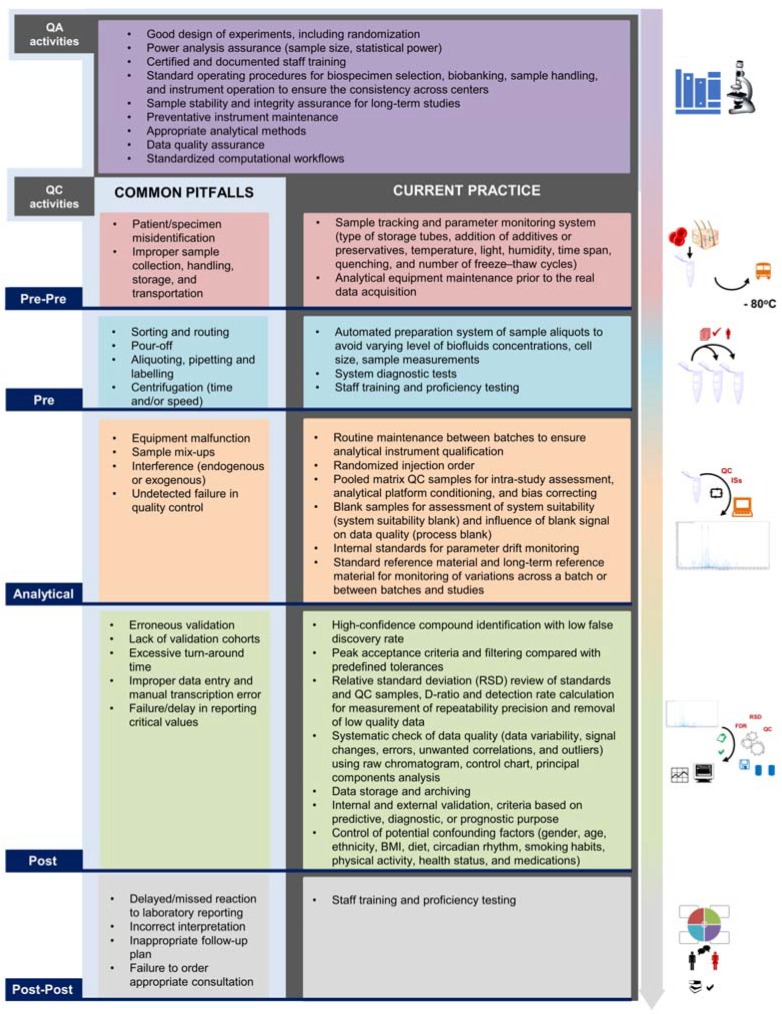
Quality assurance (QA)/quality control (QC) procedures following the novel five-step classification: pre-pre-analytical, pre-analytical, analytical, post-analytical, and post-post-analytical phases. QA activities are considered before while QC activities are undertaken during and after sample collection. The first column of QC is based on the content of five-step laboratory errors suggested by Plebani et al. [67]. The second column displays current QC techniques and activities recommended to be carried out in clinical metabolomics.

Quality assurance (QA) and quality control (QC) are two fundamental quality management procedures ensuring the success of any omics studies. At clinical and epidemiological scale, QA activities in metabolomics study are established to guarantee that data are managed by good clinical and epidemiological practice, and that the study fulfills predetermined requirements for quality. QC activities are undertaken within the QA system to verify the fulfillment of all quality requirements [58]. Existing guidelines, e.g., “Guidance for Industry: Bioanalytical Method Validation” from the Food and Drug Administration, provide QA/QC processes, which may be well adapted to targeted and semi-targeted metabolomics assays. However, they were not designed with metabolomics in mind, not routinely applied, and not readily translated into untargeted metabolomics [58]. QA/QC protocols should be ready as early as at the hypothesis formation and experimental study design step. The implementation of different QA/QC processes has been reported for MS-based metabolomics and lipidomics yet varies in both fields [58,59,60,61]. The lack of well-established QA/QC procedures for untargeted metabolomics may impede the harmonization across laboratories and multi-center studies [62]. Reducing heterogeneity across all sampling sites involves sample selection and preparation, constant cleaning of instruments, calibration, or maintenance, quality checking, data inspection at predetermined time points, and regular personnel training [48,63]. Furthermore, patient-specific variability should be raised and controlled instantly in the study design step, including, but not limited to, age, gender, ethnicity, body mass index, diet, circadian rhythms, smoking habits, physical activity, health status, and concomitant medications [64]. Taken as an example, excess exposure to glucocorticoids, which are drugs that are strictly associated with metabolic control, may affect plasma lipidome [65]. Experts in metabolomics and QA/QC procedures gathered in a meeting entitled “Think Tank on Quality Assurance and Quality Control for Untargeted Metabolomic Studies” to identify appropriate reporting standards of QA/QC activities for publications and databases, leading to the foundation of the metabolomics Quality Assurance and Quality Control Consortium [62,66]. Pioneering scientists among those who participated in the Think Tank meeting collaborated to publish guidelines that focused on the use of system suitability and QC samples in MS-based untargeted metabolomics to collect high-quality data [58]. Developing from the traditional three phases (pre-analytical, analytical, and post-analytical), we will initiate a model adopting two more steps “pre-pre-” analytical, associated with the initial sample collection, handling, and storage; and “post-post-” analytical, associated with the final interpretation to the end-users such as patients, readers of the publications, or the whole public, which have been proven to be more fallible than pre- and post-analytical actions [67]. In metabolomics, QA/QC can cover all activities from pre-pre-, pre-analytical, analytical to post- and post-post-analytical steps to ensure the acquisition of high quality and analytically reproducible data in any high-throughput study center. At clinical scale, metabolomics deals with thousands of samples, which urgently requires an appropriate experimental design and a universal QA/QC protocol. Concerning major factors that have been suggested, we will abridge current challenges based on the suggestion of Plebani et al. as well as provide firm recommendations in Figure 2 [58,67]. However, more efforts should be made to improve the current practice of metabolic phenotyping research. For instance, it is of importance to establish a platform to enhance the interpretation and comprehension of users (post-post phase) such as clinical doctors and public health specialists. Not limited to metabolomics, conventional protocols and current practice are being diversified among lipidomics laboratories, according to a recent comprehensive survey [68]. While diversity is a driving force for development, standardization and harmonization are extremely important to provide a robust outcome. The impact of this heterogeneity on metabolite and lipid quantitative measurement is currently unclear and needs to be explored further. Subsequently, all laboratories, regardless of experience, should collaborate to define community-level best practice guidelines for executing metabolomics and lipidomics experiments. This can be achieved by conducting interlaboratory studies, organizing symposiums, workshops, training sessions, or creating laboratory networks/focus groups aimed at resolving methodological issues. Another concern is the pressing need to extend the depth of metabolome coverage to prevent missing data and loss of valuable metabolites, and save cost [69]. Hence, the reference range for various kinds of biofluids and samples should be established. Once the reference interval of commonly detected metabolites is established and validated at an interlaboratory scale, it will benefit the QA/QC process and help orientate and validate other methods in the future. Additional recommendations that may be beneficial for metabolomics study are given in Table 2.

### 3.3. The Role of Establishing an Automated System for Sample Handling, Data Acquisition, and Automated Modeling

The shortage of standardized methods for sample preparation prior to analysis has been observed in metabolomics. Sample collection and processing is a major logistical problem and the largest source of errors in laboratory medicine, affecting downstream analyses [74]. Evidence-based practices to minimize pre-analytical mistakes are urgently needed and have been developed based on conventional biochemical assessments, but more research is required to determine the best method for omics techniques, including metabolomics [75]. Moreover, some metabolites are stable, whereas others are very susceptible to perturbations and improper sample handling [56]. In the clinical and epidemiological research settings, the establishment of an automated or semi-automated system for sample handling, acquisition, and automated modeling is needed to ensure robust assay performance regardless of the experience of the operator. To overcome previous methods that still necessitate some manual intervention, a fully robotic sample preparation program for LC-MS bioanalysis has recently been proven to have significantly higher efficiency, with more sample throughput, superior data quality, and promising bioanalytical time- and cost-savings [71]. Laboratory automation, by lessening hands-on activities, also minimizes possible human errors [57]. In turn, automated sample preparation still poses a considerable challenge through the additional cost and time spent on instrument calibration and software validation for robotic liquid handlers before implementation. It will get harder to figure out errors or recognize them right after they occur when the system becomes more automated [57]. Moreover, the reliability and stability of metabolites in human biofluids against all the various factors of sampling, storage, and use should be measured and known before any further applications.

### 3.4. The Assessment of Systematic Errors in Clinical Metabolomics Studies

Systematic errors of data acquisition are current challenges in MS-based metabolomics; especially, these are more problematic in the big data era because secondary data analysts may not have access to knowledge about potential data artifacts. For example, metabolomics datasets are susceptible to measurement errors caused by the timing of sample collection and decrease in instrument sensitivity [76]. However, if meta-data are not included in the dataset, the analyst cannot accurately assess the impact of these errors. Possible causes of batch effects, another type of systematic error, involve differences in instrument performance, sample handling, differences in the preparation of batches, and other unwanted environmental and technical errors [77]. Unadjusted batch variation, even subtle, may predispose to the unexpected correlation of significant features with only the batches and tend to positively bias cross-validation results [78,79]. Correction of batch variation becomes particularly essential when analyzing or meta-analyzing datasets to both increase robustness and improve reproducibility across different metabolomics studies. Data normalization for within-batch and properly between batches can be divided into three categories: data-driven normalization, internal standards-based normalization, and QC-based normalization, but standard protocols are still lacking. Several different algorithms are available to perform the batch correction, among them the common principal component analysis (CPCA) combining with median fold change was reported to perform better than solely CPCA, component correction, median fold change, or ComBat [80]. However, how to normalize data from various sources for integration purposes is still challenging, requiring additional work on data processing. Scientists need to regularly test the instrument performance and employ QC samples to adjust systematic errors and estimate variations between analytical methods [58]. QC-based normalization is becoming more popular since it regresses unwanted variation, but retains essential biological variation of interest [77]. There are multiple types of QC samples corresponding to numerous purposes, as shown in Figure 2 (the analytical step). The use of QC samples for batch correction can lead to good results when enough quality controls, e.g., one QC sample for every five biological samples, are available [81]. However, a small but quite common practice is that only one or two pooled QC samples are prepared in each batch of analysis, which may introduce another source of error since the evaporation of the employed organic solvents over time may be significant in the analysis involving many samples. Regarding computational solutions, the new version of the statTarget package with a user-friendly graphical interface integrating a QC-based random forests signal correction algorithm has been released to remove inter- and intra-batch variations in omics study [82]. NormalyzerDE, an R package, provides 12 different normalization methods for omics data to enable researchers the correct technical variation, and decide the most suitable strategy for particular datasets [83]. NOREVA is another powerful online tool that implements various normalization methods of which five well-established criteria were supported for the comparison of different normalization approaches [84]. Future research should focus on whether these adjustment methods introduce unwanted effects, such as the difference between the feature matrix and identified matrix, variations in the protocol used for QC samples analysis and the number of QC samples. Of note, batch-effect removal seems not beneficial when the batch variation is incompletely or improperly corrected, or batch-group design is unbalanced [85].

### 3.5. The Advancement and Limitations of Metabolite Annotation/Identification Standards

Protein misidentification is still a problem affecting reproducibility in proteomics, a field that is known to be more mature than metabolomics; then, it goes without question that misidentification will continue to be a bottle-neck in the more problematic area such as metabolomics [86]. Misidentification can induce false positive and/or false negative results during translation. Generally, the chemical analysis working group defined four different levels of confidence in metabolite identification observed in published metabolomics literature. Level 1 (identified compounds) requires comparison with more than one orthogonal property of a chemical reference standard acquired within the same laboratory and same technical setting. Level 2 (putatively annotated compounds) relies on the physicochemical property or spectral similarity to a known metabolite while level 3 (putatively annotated compound classes) relies on the physicochemical property or spectral similarity to those of a known chemical class of metabolites. Level 4 is about unknown compounds, but can still be extracted and quantified based on spectral data [87,88]. Lipidomics encounters a similar problem yet has distinct characteristics as discussed later in this text. In case known and unknown identification features are used together for the statistical analysis, proper information on these features, e.g., exact mass, MS/MS spectrum, retention time associated with the mobile phase, and its correlation with other elements, should be given to facilitate the later identification process. It is recommended that all authors report the level of confidence, common name, and structural code in their publication and submission to repositories. However, the employment of these standards still has not received adequate attention in peer-reviewed publications [89].

Matching the identified feature with an authentic chemical standard run on the same equipment and analytical method as the experimental samples or with MS/MS spectral databases is currently considered as the gold standard, which is impractical to interpret manually in case of enormous datasets. The major hindrance in metabolomics has now switched from identifying known MS/MS spectra molecules to the unknowns that cannot be found in the databases or that are available but do not contain experimental MS/MS data [90]. In-house spectral databases storing reference spectra have been intensively reviewed, such as The Human Metabolome Database (HMDB) and MassBank [91]. Another concern is that none of the existing metabolomics analysis software enables semi-automated search against public data in metabolomics repositories. Imitating the model of WebBLAST server for public sequencing data, a web-based MS/MS spectra data search engine called MASST has been introduced. MASST will allow users to search against all shared data on three central repositories (Metabolomics Workbench, MetaboLights, and GNPS) by converting data to a uniform open format and providing all library hits including the origin of the matched MS/MS spectral data, all dataset matches, and sample information or other metadata associated with that dataset [92]. This unique approach, called meta-annotation, will significantly improve the precision of metabolite annotation. Of note, a recently developed tool Peak Annotation and Verification Engine (PAVE) based on isotopic labeling and computational analysis indicated that only around 4% of all LC/MS peaks are annotated as putative metabolites [93].

### 3.6. Ongoing Efforts for a Universal Workflow Covering all Computational Steps of Metabolomics Research

The metabolomics community is initiating a great effort to arrive at a universal metabolomics tool that can simultaneously handle data preprocessing, processing, and visualization, as well as classification, network enrichment, and integrative analysis. Having such a platform would improve the depth of upcoming studies effortlessly and improve the reproducibility of the results. MZmine 2, MS-DIAL, and the “all-in-one” cloud-based platform of XCMS/METLIN and its variants including XCMS-MRM are among the most typical examples of the openness and complement of computational pre-processing procedures in metabolomics [90,94,95,96,97,98,99,100,101,102]. Notwithstanding, despite being the most popular statistical analysis tool used by the omics community, it still lacks functionality and is not particularly suitable for large-scale clinical metabolomics studies. Recently, developers released MetaboAnalystR 2.0, which additionally implemented raw spectral processing and functional interpretation in the endeavor to handle large datasets, support end-to-end, and to report reproducible analysis of the data [103]. Among these integration efforts, potential universal solutions have evolved but are currently under development, such as Workflow4Metabolomics, PhenoMeNal, and MetaboFlow [104,105]. Workflow4Metabolomics is based on the Galaxy environment and provides multiple metabolomics processing workflows from raw data to metabolite annotation while PhenoMeNal and MetaboFlow offer an extensive suite of open-source, standardized, interoperable, and sharable metabolomics data pipelines as a complete data analysis solution. Galaxy is an endeavor toward harmonizing and standardizing metabolomics workflows, and is much more familiar with scientists than any other platform [106]. However, these initial achievements have not reached expectations, and their influence on the metabolomics community has been unaspiring.

For further data processing and model development, missing data may introduce unintentional bias. Imputation approaches have been reported in metabolomics studies, yet many of them borrow ideas from the field of microarray gene expression [107,108,109]. Dedicated to metabolomics, an integrative model employing two unique characteristics of metabolomics data, the known metabolic network information and the relationship between features within a metabolite such as mass-to-charge ratio (*m/z*) and retention time, was generated for missing data imputation [110]. A novel multiple factor analysis framework has been developed for multi-omics studies, which estimates and imputes plausible values to the missing data by integrating information from multiple layers [111].

## 4. Specific Notes on Clinical Lipidomics Studies

In lipidomics, there have been publications, from an epidemiological perspective, investigating lipid panels of human diseases. As in other omics fields, machine learning has also gradually proved its robust role in lipidomics studies, illustrated by its application in identifying the plasma lipidome for obesity prediction in a large population-based cohort [112]. However, these current measures do not depict the entire spectrum of the human plasma lipidome [10]. Despite remarkable progress in mass spectrometric technologies, there is still a lack of consensus in lipidomics measurements between different laboratories, and the quality of lipidomics output has not satisfied the criteria of common laboratory diagnostics [113]. In this context, an initial workflow with agreed recommendations has been recently suggested to facilitate the translation of quantitative MS-based lipidomics data into clinical settings [114]. Furthermore, 31 independent laboratories jointly participated in an interlaboratory study with the purpose to discover current gaps and harmonize lipid measurements across the lipidomics community [115]. Following this interlaboratory test, the consensus location estimates for several lipid categories were robustly established, which can serve as references for quality control and quality assessment. The Lipidomics Standards Initiative Consortium has also fostered lipidomics researchers to work closely to develop common minimum acceptable guidelines and standards toward harmonized lipidomics data [116]. Lipidomics is facing the same issues as metabolomics, though some of these aspects are technically and computationally different. Excellent discussions on the technical aspects of lipidomics have occurred [113,117,118]. We hereby discuss two significant lipidomics challenges that appear to be technically divergent from typical metabolomics.

### 4.1. QA/QC and Batch Effect Removal for Large-Scale Lipidomics Studies

As mentioned before, batch effects can alter the true biological signal. Several pioneering works have attempted to capture the unique characteristics of batch effects adjustment in lipidomics research. The population-based study within the Alzheimer Disease Neuroimaging Initiative cohort is a typical example of the role of QA/QC and technical variance removal in large-scale lipidomics studies. The authors employed QC samples to verify the high reproducibility of lipidomics data and the relative standard deviation (RSD) in QC samples as an inclusion criterion to establish the final lipid dataset for further statistical analysis [119]. In the subsequent data processing, they used the QC-based LOESS normalization method for batch effects adjustment to minimize the analytical variations [119]. In addition, a novel QC-based correction method using random forests (called SERRF) has been developed. This method outperformed other commonly used normalization methods and was reported to have lowered unwanted systematic errors to 5% RSD in a large-scale lipidomics study [120].

### 4.2. The Need for High-Confidence Lipid Identification and Quantification

The top five challenges raised by the lipidomics community were (1) the shortage of standardization of methods and protocols, (2) the lack of reference standards, (3) the lack of data handling and quantification software, (4) over-reporting, and (5) the issue of false positives [68].

In lipid identification, level 1 of confidence is mostly not possible for a large number of lipid species. Fortunately, similar to peptides, the MS/MS patterns of significant lipid classes are predictable, which makes the in silico annotation and prediction of major lipids possible when consulting the MS/MS with biologically plausible fatty acid chains. Noticeably, the identification of lipid subclass compositions such as phosphatidylcholines (PCs), lysoPCs, and sphingomyelins at the fatty acid chain resolution using the AbsoluteIDQ^TM^ p150 kit and Lipidyzer^TM^ platform provides evidence of the odd-chain fatty acids in PCs, which is quite essential and acknowledged. This method or other similar ones may be used in large-scale lipidomics research [121]. In-source fragmentation generates precursor ions whose masses are similar to those of endogenous lipids, which may induce misannotation. Therefore, a test for in-source fragments is recommended as an essential step in lipidomics method development [122]. Moreover, the advancement in separation techniques (e.g., ion mobility) and resolution of the mass spectrometer for lipid identification is needed to differentiate between isobaric species belonging to different lipid categories, resolve isotope effect, as well as allow researchers to directly quantify them in total lipid extracts [123].

Under the Lipidomics Standard Initiative, good practices and recommendations toward the standardization of crucial steps in lipid species identification have been discussed [124,125,126]. Concerning in silico lipidomics libraries, the major ones include LIPID MAPS and LipidBlast, followed by prospective executable packages such as MS-DIAL, LipidMatch, and LipiDex. In silico libraries allow users to identify lipids through a broad set of high-resolution tandem mass spectrometry experiments, hence improving biological interpretation [127,128,129]. Following the fast progress in lipidomics over the last two decades, new tools are needed to get more insights into this field. An R-based, open-source tool called Lipid Mining and Ontology (Lipid Mini-On) was later developed, which can split individual lipids into multiple ontology classes with Lipid Ontology terms (similar to Gene Ontology) based on LIPID MAPS classification as well as other molecular characteristics for the enrichment analysis of lipids currently absent from databases [130]. LION, a new and freely accessible lipid ontology, is able to assign more than 50,000 lipid species to various biophysical, chemical, and cell biological processes [131]. This web-based resource with a complementary enrichment analysis tool empowers researchers to investigate the complex lipidomes in biological systems.

## 5. Toward Reproducible Data Analysis and Interpretation in Clinical Metabolomics and Lipidomics

One of the possible future directions of computational metabolomics platforms is to diversify and standardize analytical processes for a variety of study designs, including cross-sectional and longitudinal studies, and clinical trials. In that context, the role of machine learning cannot be ignored. Machine learning has the power to process a large amount of data that exceeds human capacity, gather lessons and experiences from nearly all clinicians, and guide patients instantly [132]. A recent study compared the performance for phenotype differentiation in untargeted metabolomics among different machine learning techniques. Concerning all aspects including skewed error distributions, unbalanced phenotype allocation, biological and technical outliers, missing data, and dimensionality reduction, the two non-linear methods, support vector machine and random forests, act as the leading algorithms over naïve Bayes, neural networks, sPLS-DA, PLS-DA, and k-nearest neighbors classifiers [133]. Additionally, the opaque methods usually yield accurate predictions but are more complicated to interpret than the transparent ones. As a solution, algorithms such as LIME and iBreakDown show promise for explaining the predictions of any complex, black-box model [134,135,136,137]. It is worth mentioning that the so-called explainable prediction models are most beneficial from the datasets that have a small number of features from input data. Feature selection/engineering in machine learning is an ongoing research topic. In that respect, automated and flexible feature engineering algorithms may be essential to increase the throughput, reproducibility, and transferability. Although several machine learning methods have been experimented, the interpretation of several complicated cases is still unfeasible and remains an open question [138]. Interestingly, an expert knowledge-collecting tool called tinderesting allows experts to rate the features, which will be subsequently used to train a machine learning model and may improve the overall performance of the prediction model in dynamic metabolomics studies [139].

Metabolomics research often recruits a small sample size, usually less than 300 per group for classification purposes, which may not ensure the study’s reliability [140]. Hence, meta-analysis has evolved as a method of choice to enhance the study’s robustness and minimize the risk of false discoveries [141]. Finally, the value of meta-analysis in metabolomics has been demonstrated to increase the probability of determining metabolites associated with the phenotype of interest [142,143]. Robust meta-analysis and network-based interaction visualization frameworks should be investigated for metabolomics datasets to bring the interpretation of metabolomics data to the next level. Of note, another method to increase the study robustness given small sample size is the application of appropriate study design such as prospective longitudinal study.

## 6. Fundamental Requirements on Standards for Reporting Metabolomics-Based Biomarker Studies at Clinical and Epidemiological Scale

The standard for reporting metabolomics-based biomarker discovery studies is still ambiguous and incomplete [144]. A thorough understanding of principles, adequate guidelines, and practical tools has minimized the pitfalls and fortified critical appraisal, validation, and replication of studies. The main characteristics of reporting principles are matching, exhaustive, skeptical, second-order, transparent, and reproducible [145]. In clinical metabolomics studies, authors are urged to report whether data appear reasonably matched to the hypothesis (data matching), whether there are alternative approaches to explore data (exhaustiveness), whether the analysis addresses any other premises or questions (skepticism), whether data pertain directly to the investigation or just contribute to relevant background (second-order), whether there are means such as data visualization or data summary included in the analysis that highlights the connection between data and results (transparency), and whether the code and data are made available in open sources (reproducibility) [145]. There are several reporting guidelines for clinical studies and multivariable prediction models, especially for diagnostic and prognostic purposes, such as the Transparent Reporting of a multivariable prediction model for Individual Prognosis Or Diagnosis (TRIPOD) statements, the Standards for the Reporting of Diagnostic Accuracy Studies (STARD), and the Biospecimen Reporting for Improved Study Quality (BRISQ) [146,147,148]. However, these guidelines are poorly incorporated in the omics field in general and in metabolomics in particular. Specifically, the assessment of metabolite and lipid biomarkers currently makes up a considerable portion of metabolomics research applications, whereas epidemiological studies have their unique features at the population level. Collectively, a reporting guideline combining biomarker diagnostic/prognostic characteristics with distinct epidemiological aspects in terms of study design, sample size, analytical approach, confounding, and bias should be developed to fully satisfy metabolomics study at clinical and epidemiological scales. The QUADOMICs tool, an adaptation for omics study of the Quality Assessment of Diagnostic Accuracy Studies (QUADAS), assesses the methodological quality of omics-based diagnostic studies, and could be considered as a prototype to help develop an appropriate guideline for metabolomics [149]. Recently, a novel reporting guideline based on R Markdown template for the data analysis step of biomarker discovery metabolomics studies has been issued [150]. The new guideline, along with our suggested one in reference to STROBE, TRIPOD, and QUADOMICs, if applied, will improve the completeness, transparency, and reproducibility of clinical lipidomics and metabolomics study (Table 3). Metabolomics journals should obligate authors to share their code as a requirement for publication rather than recommend it.

## 7. Multi-Omics Integration for Precision Medicine

The exponential increase in amount of data in association with the gradual rise in sample numbers is the de facto characteristic of omics sciences. Single omics analysis usually reveals correlations of reactive processes rather than causations [153]. Years of constant endeavors have proved that focusing on technological “silos” is incapable of finding solutions to complex molecular pathophysiological issues [154]. It is undeniable that metabolomics itself brings substantial advantages to the multi-omics system, particularly in the diseases that have dysregulated metabolism, such as diabetes and cancer. For instance, in the predictive model of stress events in prediabetes patients, the classification performance of metabolomics was only overshadowed by that of multi-omics with the area under the receiver operating characteristic curve, reaching 80.1% [155]. The integration of multi-omics data, however, has the power to explain potential causative changes, strengthen the contribution of omics science to our understanding of biomedicine, and provide a complete picture of diseases [156]. In the context of data explosion at every omics level and accumulated knowledge of complex interactions between those molecular layers, a better paradigm integrating multi-omics is a potential solution to comprehend the underlying biological processes as well as to give better predictions on the outcome of interest (e.g., diagnosis, prognosis) [157,158,159]. For this exact reason, the idea of a single platform for a unifying proteomics-metabolomics analysis has been proposed [160]. We hereby provide some excellent examples to demonstrate the potential of multi-omics approaches. The combination of multi-omics technologies is undeniable in investigating cerebrospinal fluid (CSF) and expanding the coverage of CSF metabolites since biomolecules are interactive to exert their functions at multiple levels in a biological specimen [161]. The combination of multi-omics with the prospective longitudinal characteristic helps reinforce the causality. An integrated network analysis of genomics, metabolomics, and Alzheimer’s disease (AD) risk factors in 1111 subjects of Wisconsin Registry for Alzheimer’s Prevention study revealed that several genes might be indirectly associated with AD risk through particular metabolites [162]. In type 2 diabetes mellitus, a rich longitudinal investigation integrating transcriptomics, proteomics, metabolomics, and microbiomics sheds light on several molecular pathways, host-microbe interactions, and responses to infections that differed between glucose-dysregulated and healthy subjects, and highlighted the diverse intra- and inter-individual variabilities [155]. Another example, the transcriptomics-metabolomics integrated longitudinal research on molecular profiles of host-microbe interactions provided the most comprehensive summary, until now, of dysbiosis state in the gut microbiome in inflammatory bowel disease [163]. Fusing genome-wide association studies information with metabolomics data permits the simultaneous assessment of the genetic (or intrinsic) and environmental (or extrinsic) effects on disease phenotypes, which is better than using solely metabolomics [164]. Recently, an in-depth, prospective, longitudinal, phenotyping study gathering information on medical history, clinical and behavioral status, genome, transcriptome, proteome, immunome, metabolome, microbiome, exposome, and imaging from 109 subjects has been developed to comprehensively determine health status and establish risk profiles of metabolic, cardiovascular, fitness-frailty, cognitive, infectious, psychological, oncological, and inflammatory diseases [165].

**Figure 3:**
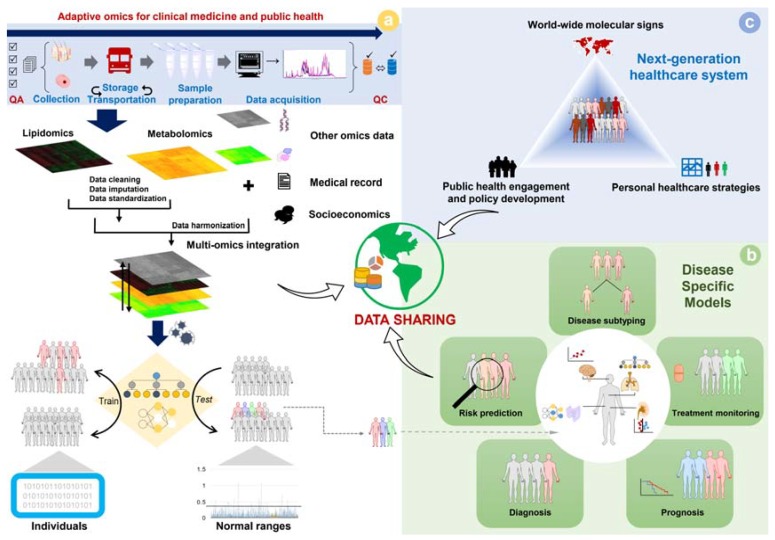
Metabolic phenotyping as a core member to establish multi-omics biosignatures for the development of the next-generation healthcare system: (**a**) adaptive workflow for a population-based study to determine the normal range of lipidome/metabolome, and detect nonspecifically abnormal conditions based on quantitative multi-omics profiles in reference to intrinsic, extrinsic, and socioeconomic factors; (**b**) corresponding models constructed for disease-specific purposes; (**c**) the next-generation healthcare system based on advances in smart engines with omics data as the primary pillar, and promoted by data sharing and machine learning.

The practicality of clinical metabolomics, as a core member of the omics-informed precision medicine system, is suggested in Figure 3. When all pitfalls are overcome, we may be able to establish a model from quantitative multi-omics data to determine the normal range of the population and is capable of detecting unspecific abnormal multi-omics profiles as seen in Figure 3A. It is essential to emphasize that the genetic background should be considered along with other socioeconomic factors, and the interactions among factors should be taken into account when constructing an omics-based model [166,167]. Combining with the advancement of omics-based disease-specific prediction, persons with a suspect medical condition will be determined for a proper intervention (Figure 3B). With the accumulation of these smart engines over time, together with benefits from data sharing of every aspect, we will be able to transform the medical system, of which omics profile is the major pillar (Figure 3C). The future of precision medicine is personomics, which is the combination of multi-omics, environmental factors, social interactions, and lifestyles (behaviors) based on unique personal references.

Once again, it is important to emphasize the extraordinary role of machine learning in advancing multi-omics studies and reshaping our healthcare system. High quality multi-omics studies should formulate proper study hypotheses and select samples considering biological matrix characteristics. Regarding data processing and analysis, they are expected to handle and store samples avoiding adverse effects caused by intrinsic and extrinsic factors, carefully collect quantitative multi-omics data and associated meta-data, and employ better integration and interpretation software. For data replication, authors should adopt minimum standards for multi-omics methods and meta-data, and develop new tools for depositing intact multi-omics data rather than splitting it into multiple single datasets [168]. Regarding this matter, considerable efforts have been made toward an algorithm and comprehensive tools for analyzing omics data at the multi-omics scale to archive the most information and eliminate the most noise [169,170,171]. Herein, three major opinions have been raised with distinct properties: the genome first, the phenome first, and the environment first approaches [153]. Future tools and software should consider the interactions between different omics layers to extend the potential of this approach entirely.

## 8. Potential Bioethical Issues Associated with the Sharing of Clinical Metabolomics and Lipidomics Data

As in genomics, the most mature of the omics fields, there are ethical concerns that are worth paying attention to when we handle metabolomics data, including, but not limited to, privacy and identifiability, harmonization across sites regarding informed consent and institutional review board, incidental findings, data ownership, and funding priority [172]. Toward clinical application, the completeness of personal information, including medical records, medication lists, and socioeconomic status, would be the major obstacle. Sharing data from research involving human samples should comply with the ethical, legal, and social implications, and patient privacy must always be protected as the first priority [173]. Nevertheless, unlike genomics, metabolomics data have a cross-sectional characteristic and are not unique to a particular individual; therefore, they do not contain factors listed in the “Safe Harbor” method for de-identification under the Health Insurance Portability and Accountability Act privacy rule. There are currently no known methods of recognizing a patient based on their metabolic panel, except potentially for rare diseases [173]. In this way, risks of identification can be avoided, which is the everlasting concern for genomics research participants who are afraid that their genomic information may be used for adversary attacks.

## 9. Perspectives and Conclusions

It is undebatable that the availability of research data and standardized report publications will substantially eradicate barriers against scientific progress. Throughout the paper, we have made considerable attempts to explore the remaining challenges, evaluate current opportunities, and suggest several future directions that should be considered by the metabolomics and lipidomics community to facilitate data acquisition, analysis, and sharing at the clinical and epidemiological scale. Physicians should be prepared to use “cognitive computers” applying artificial intelligence and machine learning, which in the near future, will be implemented in the hospital settings to assist in disease diagnosis and prediction of patient outcomes. In the big picture, metabolomics and lipidomics research has to be allied to a network of multi-layer omics combining with the power of machine learning, artificial intelligence, and standardized QA/QC protocol. We are also pioneers in the suggestion of a complete framework for quantitative metabolomics research strictly based on standardizations and reporting guidelines from study design to interpretation to the end-users. On the whole, the metabolomics community is encouraged to be involved in the joint multi-omics platforms, e.g., The Cancer Genome Atlas and International Cancer Genome Consortium, to facilitate the advancement of molecular research and precision medicine. International and multidisciplinary efforts are required to achieve this goal. Finally, although we did not intensively go through related bioethical issues in this review, they should always be taken into account to complement the next-generation healthcare system.

## Figures and Tables

**Table 1:** Recent representative achievements of metabolomics in public health and clinical research.

Country	Year	Metabolomics/Lipidomics/Multi-omics	Clinical Field	Platform	Main Conclusions	References
The United Kingdom	2011	Steroidomics	Cancer	GC-MS	This study combined population-based steroidomics research with machine learning analysis, aiming at identifying urine steroid biomarkers for differentiating adrenocortical carcinoma from benign adenoma.	Arlt et al. [27]
The United States	2011	Metabolomics	Diabetes	LC-MS	A large nested group of 2422 normoglycemic subjects in the Framingham Offspring Study was followed for 12 years to help identify a panel of three amino acids (isoleucine, phenylalanine, and tyrosine) which might serve as novel predictors for future diabetes.	Wang et al. [28]
The Netherlands	2015	Steroidomics	Cancer	GC-MS	Urinary steroid signatures were established for the discrimination of adrenal cortical carcinoma from other adrenal conditions.	Kerkhofs et al. [29]
The United Kingdom	2015	Metabolomics	Physiological state	GC-MS and LC-MS	The Husermet project has applied untargeted MS to investigate the comprehensive hydrophilic and lipophilic metabolome of serum biospecimen obtained from the phenotyping of 1200 healthy subjects.	Dunn et al. [30]
The United States	2015	Steroidomics	Cancer	LC-MS	The novel LC-MS/MS assay used in this study enabled the examination of all estrogen metabolites for epidemiological and clinical research on hormone-related diseases.	Ziegler et al. [31]
Multinational	2018	Multi-omics	Diabetes	N/A	The Environmental Determinants of Diabetes in the Young (TEDDY) study followed more than 12,000 children to explore metabolic pathways related to type 1 diabetes.	Rewers et al. [32]
Multinational	2018	Multi-omics	Obesity	NMR and LC-MS	Along with fecal metagenomes, plasma and urine metabolome revealed molecular pathways uniting the gut microbiome and the human phenome to hepatic steatosis in two cohorts of non-diabetic obese women in the FLORINASH consortium.	Hoyles et al. [33]
Japan	2018	Metabolomics	Physiological state	CE-MS (and LC-MS)	The Tsuruoka large-scale cohort study has gathered plasma metabolomics data from more than 10,000 individuals as an innovative model for preventive medicine.	Harada et al. [34]
Multinational	2019	Metabolomics	Long-term mortality risk	NMR	From the metabolic profile of 44,168 individuals, 14 biomarkers have been associated with all-cause mortality. The combination of this set of metabolite predictors and sex considerably improves mortality risk prediction compared to traditional risk factors such as age, body mass index, systolic blood pressure, or total cholesterol.	Deelen et al. [35]
The United Kingdom	2019	Metabolomics	Parkinson’s disease	TD-GC-MS	This study applied an unbiased method, taking the use of volatile sebum metabolites to diagnose Parkinson’s disease.	Trivedi et al. [36]
The United Kingdom	2019	Metabolomics	Cardiovascular disease	NMR	This study with over 7000 participants on the metabolic profile of atherosclerosis revealed that this condition was associated with perturbations of multiple interconnected pathways related to lipid, fatty acid, and amino acid metabolism, and displayed a considerably similar model between coronary and carotid atherosclerosis.	Tzoulaki et al. [37]
The United States	2019	Metabolomics	Obesity	LC-MS	This study revealed the disturbance of the metabolome in obese versus healthy individuals. Approximately a third of compounds followed changes in body mass index, suggesting the role of metabolome profiling in participant recruitment for clinical trials related to obesity.	Cirulli et al. [38]
The United States	2019	Metabolomics	Diabetes	LC-MS	A total of 69 out of 331 plasma metabolites extracted from more than 2000 samples from the Diabetes Prevention Program were linked to type 2 diabetes regardless of treatment randomization.	Chen et al. [39]
Sweden	2019	Metabolomics	Physiological state	LC-MS	Not just stopping at the cross-sectional quantification of urinary eicosanoid metabolites, this recently published study extended the scope by focusing on the long-term repeatability and stability of the method used in order to serve the large-scale analysis of multiple cohorts.	Gomez et al. [40]

**Table 2:** Additional recommendations to ensure QA/QC procedures in metabolomics and lipidomics study at clinical and epidemiological scale.

Phases ^1^	Recommendations ^2^
**Pre-pre-analytical**	- Need of universal and reproducible study protocols for different tissue and biofluid types to reduce the effects of extrinsic exposures (e.g., enzymatic activities, oxygen, UV light, or temperature) and intrinsic factors (e.g., age, gender, health status, body mass index, circadian rhythm), or to standardize exposure of samples to these effects when inevitable. - Need for well-managed metabolomics biobanks with the proper collection, processing, storage, and tracking processes. - Need for an automated system for sample handling and preparation. - Consultation with an experienced statistician in the omics field.
**Pre-analytical**	- Application of a single validated standard operating procedure (SOP) for the pre-analytical phase across all sampling sites. - Need of a standardized protocol to preserve samples, avoid prolonged storage at room temperature and multiple numbers of freeze and thaw cycles. - Assessment of the suitability of the analytical platform using system suitability samples and blank samples. - Personalized training and education, different for novice and experienced researchers.
**Analytical**	- Adoption and calibration of every apparatus used under a quality assurance system, verification for unexpected variations, and routine maintenance. - Need for a pooled QC sample or when impossible, an alternative QC sample from the first batch of samples randomly collected. - Need for reporting standards/SOPs (at every step of the analytical process). - Establishment of minimum acceptance criteria for analyzed samples.
**Post-analytical**	- Establishment of a database of authentic standards using the same analytical condition to prevent misidentification (e.g., Fiehn’s library that has more than 1000 authentic standards). - Use of data visualization tools to evaluate the analytical run quality and check for systematic and random errors. - Establishment of a strategy for statistical modeling. - Consideration of blindness if needed.
**Post-post-analytical**	- Report of QC metadata (e.g., sample order, QC sample, reference materials used). - Education of the community about QC procedures. - Standardized data sharing on a public repository. - Personalized training and education.

Note: ^1^ Based on the content of five-step laboratory errors suggested by Plebani et al. [67]. ^2^ In reference to [49,58,62,70,71,72,73].

**Table 3:** Proposed checklist for clinical metabolomics-based biomarker discovery and validation in reference to STROBE, TRIPOD, and QUADOMICs.

Section/Topic	Item	Essential Topic	STROBE [151]	TRIPOD [146]	QUADOMICs [149]	Our In-House Assessment [143]	Checklist Item ^1^
**1. Title and abstract**							
Title	1a	Yes	1	1			Specify the study design with developing and/or validating purpose, the target population, and the outcome with simple and straightforward terms in the title.
Abstract	1b	Yes	1	2			Depending on the target journal, provide in the abstract a precise and structured summary of objectives, study design, study participants, sample size, type of samples (e.g., plasma, serum, or urine), analytical platform, predictor variables, outcome, statistical method, results, and conclusions.
**2. Introduction**							
Background	2a	Yes	2	3a			Provide the scientific and clinical background (including the diagnostic or prognostic purpose) and explain the rationale for developing and/or validating the multivariable prediction model, in regard to existing models.
Objectives	2b	Yes	3	3b			Determine the objectives and hypotheses, emphasizing if it is a development and/or validation research of the model.
**3. Methods**							
Ethical approval	3a	Yes					Clearly report ethics committee approval and participant consent.
Study design	3b	Yes	4	4a		Item 1	State the study design (e.g., case-control, cohort, randomized trial, or registry data) or source of data (e.g., biobank, public database).
Setting	3c	Yes	5	4b			Describe the settings, locations, and relevant dates where the data were collected, including periods of follow-up, if applicable.
Participants	3d	Yes	6	5a–5c	Item 1 and 2	Item 2–6	(a) Cross-sectional study: Give the inclusion and exclusion criteria, and the sources and methods of recruitment of study participants. (b) Longitudinal study: Give the inclusion and exclusion criteria, and the sources and methods of recruitment of study participants. Describe follow-up methods. Give diagnostic criteria and prior treatment, if applicable. For developing purpose, the spectrum of patients should represent those who will receive the test in practice.
Sample size	3e	Yes	10	8			Explain how the sample size was determined.
Sample collection	3f	Yes			Item 3 and 4	Item 8	Describe the type of samples, the procedures and timing of biological sample collection with reference to clinical factors and the methods to control metabolome changes (e.g., arterial versus venous blood, circadian oscillations, pre- and post-prandial status, the time between sampling and storage).
Sample storage	3g	Yes			Item 5	Item 8	Describe the methods to control chemical and enzymatic degradation and/or interconversion.
Sample preparation	3h	Yes			Item 5	Item 9	Describe the methods to control analytical errors and between-batch variations.
Data acquisition	3i	Yes				Item 10–13	Describe the experimental conditions, the analytical validation methods, and the number of batches of analysis.
Data preprocessing and treatment	3j	Yes				Item 15	Report parameters for peak detection (i.e., algorithms and acceptance criteria for valid peaks), deconvolution, alignment, and correction. Describe the methods to filter data noise, impute missing values, and correct batch effects (e.g., data-driven, internal standards-based, or QC-based normalization).
Predictors	3k	Yes	8	7a–7b			Define all variables and explain their measurements used in constructing the multivariable prediction model.
Bias	3l	Yes	9				Characterize any attempts to show and solve potential sources of bias.
Missing data	3m	Yes	12	9			Describe how missing data (e.g., samples) were handled.
Metabolite identification ^2^	3n	Yes				Item 14	Describe the methods for metabolite identification and the level of confidence of identified compounds. Report whether a match with authentic standards has been conducted.
Multi-omics data integration	3o	Optional					Report the multi-omics data integration, if available. Description of integration strategies, such as post-analysis data integration or simultaneous integration from different omics data types, is recommended.
Statistical analysis and modeling	3p	Yes	12	10a–10e and 12	Item 16	Item 16	Describe statistical methods, the methods to detect outliers, validation methods, and performance measures (e.g., AUC, accuracy, sensitivity, and specificity). Describe any updates of the model after the validation, if done. For the validation purpose, determine any inconsistencies from the development data.
Outcome	3q	Yes	7	6a–6b			Explain the assessment of outcome that is predicted by the multivariable prediction model. Report any efforts to the blind assessment of the predicted outcome.
**4. Results**							
Participants	4a	Yes	13	13a–13c			Report numbers of study participants at each stage (e.g., numbers potentially eligible, screened for eligibility, validated eligible, included in the study, finishing follow-up, and analyzed) and the number of participants with missing information for predictors and outcome. Give reasons for non-participation or exclusion at each stage.
Descriptive data	4b	Yes	14	13a–13c			Designate characteristics of study participants (e.g., baseline demographic, clinical, and socioeconomic status) and information on potential confounding factors.
Data exploratory analysis	4c	Yes					Report exploratory data analysis (e.g., using unsupervised learning approaches).
Model development	4d	Yes	15–16	14a–14b			Specify the number of participants and outcomes in each analysis. Report the unadjusted and adjusted potential confounders that may influence associations between each predictor and outcome.
Model interpretation	4e	Optional	15–16	15a–15b	Item 14 and 15		Present the full prediction model to enable reproducibility for individuals. Explain how to interpret the prediction model in a human-friendly approach (e.g., LIME, iBreakDown). In case uninterpretable, indeterminate data should be reported.
Model performance	4f	Yes	15–16	16			Report measures of performance of the prediction model. Describe the effects of unbalancing (e.g., cases versus controls) to the performance of the model.
Reference standards comparison	4g	Optional			Item 6–13	Item 7	Compare the constructed models with currently approved approaches (e.g., CA 19.9 for diagnosing pancreatic cancer).
Model updating	4h	Optional	17	17			Report the results of any updates of the model. An updated model using quantitative information of the tentative biomarkers are strongly recommended.
**5. Discussion**							
Key results	5a	Yes	18				Summarize critical results in harmony with study objectives.
Interpretation	5b	Yes	20	19a–19b			Give an overall evaluation of results based on study objectives, results from previous similar studies, and other related evidence. For validation purpose, discuss the results in regard to the performance of development data, and any other validation data from the public databases.
Limitations	5c	Yes	19	18			Discuss the limitations of the study, considering sources of potential confounders, biases, and statistical uncertainty.
Implications	5d	Yes	21	20			Discuss the potential application of the model into clinical settings and suggestions for future research and practice.
**6. Other information**							
Supplementary materials	6a	Yes		21			Provide available supplementary files, such as the full study protocol and generated datasets.
Funding	6b	Yes	22	22			Give the sources of funding and the influence of each funder on the outcome and, if applicable, for the original study from which the current paper arises.
Conflicts of interest	6c	Yes					Clearly declare potential conflicts of interest.
Repositories for generated data	6d	Yes					Report the public repository for generated data concerning FAIR (Findability, Accessibility, Interoperability, and Reusability) principles ^3^.
Executive commands	6e	Optional					Report any programming code used (e.g., R code).

Note: ^1^ The description was adopted with modifications from STROBE, TRIPOD, and QUADOMICs guidelines along with our in-house assessment. ^2^ Metabolite identification step can be performed prior to or after the Statistical analysis and modeling step. ^3^ In reference to [152].
